# The standards for recognition of occupational cancers related with polycyclic aromatic hydrocarbons (PAHs) in Korea

**DOI:** 10.1186/s40557-018-0224-1

**Published:** 2018-02-26

**Authors:** Tae-Won Jang, Yangho Kim, Jong-Uk Won, Jong-Seong Lee, Jaechul Song

**Affiliations:** 1 Department of Occupational and Environmental Medicine, Hanyang University College of Medicine, 222, Wangsimni-ro, Seongdong-gu, Seoul, 04763 South Korea; 2 Department of Occupational and Environmental Medicine, Ulsan University Hospital, University of Ulsan College of Medicine, 877, Bangeojinsunhwando-ro, Dong-gu, Ulsan, 44055 South Korea; 3 Department of Preventive Medicine, Yonsei University College of Medicine, 50, Yonsei-ro, Seodaemun-gu, Seoul, 03722 South Korea; 4 Center for Occupational Lung Diseases, 478, Munemi-ro, Bupyeong-gu, Incheon, 21417 South Korea

**Keywords:** Cancer, Occupation, PAHs

## Abstract

Polycyclic aromatic hydrocarbons (PAHs) are organic compounds containing carbon and hydrogen. PAHs have carcinogenicity in human. Cancers related with PAHs include cancers of lung, skin, bladder, and others. International Agency for Research on Cancer (IARC) has determined several occupations that can be exposure to PAHs were probable carcinogens to human. National Toxicology Program (NTP) classified coal tars and coal tar pitches, and coke oven emissions as known to human carcinogens, and US Environmental Protection Agency (EPA) classified coke oven emissions as human carcinogen.

PAHs can be produced both naturally and artificially. Sources of occupational exposure include coal gasification, coke production, coal tar distillation, aluminium production, and so on. Diesel exhaust emission contains large amount of PAHs. Cigarette smoking also contains many PAHs, which is the important source of environmental source of PAHs.

The evaluation for work-relatedness and standards for recognition of occupational cancers should focus on occupations that can be exposed to PAHs. In Korea, standards for recognition of occupational cancers related with PAHs are following: lung cancers related with more than 10 years exposure to coal tar pitch, lung and skin cancers related with soot exposure, and skin cancers related with more than 10 years exposure to coal tar. When applying these standards, occupations that can be exposed to PAHs should be focused on. In addition, latent period for solid cancer should be considered. In addition to these occupations, diesel engine combustion and firefighters can be exposed to PAHs.

## Background

Polycyclic aromatic hydrocarbons (PAHs) are organic compounds containing carbon and hydrogen. PAHs are composed of multiple aromatic rings. They are derived naturally from coal and tar deposits, and produced by incomplete combustion of organic matter. They are contained in vehicle exhausts and tobacco smoke, which are important sources of environmental pollution.

Many researchers reported PAHs have carcinogenicity in human, and induce many cancers. Cancers related with PAHs include cancers of lung [1–5], skin [4, 5], bladder [1–5], breast [6, 7], and stomach [5] cancers. International Agency for Research on Cancer (IARC) [8] has reviewed the studies about carcinogenicity of PAHs and determined PAHs were probable carcinogens to human. National Toxicology Program (NTP) has reported that 15 individual PAHs were reasonably anticipated to be human carcinogens based on sufficient evidence of carcinogenicity from studies in experimental animals [9].

Standards for recognition of occupational diseases are regulated in the Enforcement Rules of the Industrial Accident Compensation Insurance Act (ER-IACIA). Standards for recognition of occupational cancers related with PAHs are also contained in this regulation. According to this regulation, lung cancers related with more than 10 years exposure to coal tar patch, lung and skin cancers related with soot exposure, and skin cancers related with more than 10 years exposure to coal tar are considered as occupational cancers.

PAHs are complex of many compounds, not single substances. For this reason, it is difficult to evaluate work-relatedness between suspicious PAHs exposure and cancers. The objective of this study is to review the epidemiologic evidences on the relationship between PAHs and cancers, and identify the information which may help to evaluate the relatedness between PAHs and cancers.

### In vivo metabolism of PAHs

PAHs enter human body through ingestion, inhalation, and dermal absorption. Absorbed PAHs enter lymph and circulate in the blood, and are metabolized in liver and excreted through bile and urine [10–12]. PAHs are also metabolized in many organs including adrenal gland, testis, thyroid, lung, skin, and small intestine [12]. PAHs accumulate in breast milk because of their lipophilic property [13]. The most widely used metabolites of PAHs is urinary 1-hydroxypyrene, which is used for biomarkers of PAHs exposure [14]. The American Conference of Governmental Industrial Hygienists (ACGIH) recommended urinary 1-hydroxypyrene in the end of work shift or end of work week as biologic exposure index for assessment of exposure to PAHs [15]. Half-life for excretion through bile and urine is 22 h and 28 h, respectively [16].

### Source of exposure

PAHs are natural component of forest fire, fossil fuels, and byproducts of combustion processes for heating and energy [17]. PAHs can be produced both naturally and artificially. Sources of occupational exposure include coal gasification, aluminum production, coke production, road pavement, and so on [8, 18]. Diesel exhaust emission contains large amount of PAHs [19]. Cigarette smoking also contains many PAHs, which is the important source of environmental source of PAHs [20].

#### Occupational exposure

For the past, chimney sweeps have been exposed to soot when they cleaned by pushing a steel brush from the top or the bottom of chimney. Soot is a by-product of burning or pyrolysis of carbon-bearing coal, wood, fuel, paper, plastic, and other organic matter. The composition and properties of soot vary depending on the type of material burned and the state of combustion. Generally, soot consists of carbon material, inorganic compounds and water-soluble organic matter. Water-soluble organic substances are substances extracted from organic solvents and contain PAHs [21].

Coal gasification is the process of producing synthesis gas. This process reacts coal with oxygen and carbon dioxide, and forms synthesis gas with hydrogen and carbon monoxide. Coal gasification process is a type of incomplete combustion, which produces byproducts containing carbon monoxide and PAHs [22]. In addition, many materials such as asbestos, silica, arsenic, cadmium, lead, nickel, vanadium, sulfur dioxide, and others are produced in coal gasification process. Integrated gasification combined cycle (IGCC) is a power generation technology using coal gasification process [23].

Coal carbonization is the process of producing coke for use in steel-making furnaces and other metal smelting processes [24]. Coal carbonization process involves heating the coal to a high temperature of 1300 degrees Celsius without oxygen to distill tar and light oil from the coal. In this process, coke oven gas is removed from coal with ammonia, water, sulfur compounds, and others. Coke oven gas consists of hydrogen, methane, carbon monoxide, carbon dioxide, ammonia, hydrogen sulfide, and tar compounds which contain many PAHs [25]. Wood gasification extracting oil and tar, which had once been used imperial Japan during World War II, is also PAH exposed process similar to coke production. Charcoal production is a wood gasification process, although it dismisses gas leaving only carbon backbone.

Coal tar is black sticky liquid, which contains phenol compounds, aromatic nitrogen bases and alkyl derivatives, paraffinic and olefinic hydrocarbons, and various PAHs [17]. Coal tar is produced by cooling coal gas to room temperature, which is called coal tar distillation process. The byproducts of coal tar distillation are classified into two types: the first is mixture of mononuclear or polynuclear aromatic hydrocarbons which are distilled by heating the coal to 400 degrees Celsius in the atmospheric pressure; the second is coal tar pitch, which is residue after distillation. Creosote is carbonaceous chemical formed by the distillation of various tars and pyrolysis of wood or fossil fuel. Creosote has been used as a wood preservative since the nineteenth century and is now used as a preservative for railway sleepers, poles, marine piles, fences, and various wood products. Creosote consists of PAHs and phenol compounds [26]. Coal tar pitch contains mononuclear or polynuclear aromatic hydrocarbons, heterocyclic compounds, and others [27].

Roofing is a process of removing and installing the roof. This process consist of removing, which is cutting, prying, and scraping old roof, and installing, which is applying liquid coal tar pitch on the surface and installing new roof. Bitumen is used instead of coal tar pitch in this process recently. Bitumen, also known as asphalt, is sticky semi-solid of black or blackish brown, which contains aliphatic hydrocarbons, aromatic hydrocarbons, polynuclear aromatic hydrocarbons, heterocyclic compounds, oxygen, sulfur, and heavy metals such as iron, nickel, and vanadium. The content of PAHs in bitumen is much lower than coal tar pitch [28].

Road paving is a process of spreading aggregates such as stones and gravel on the road and rolling it up with a roller. In this process, coal tar pitch is mixed with aggregates and spread on the road. Since coal tar pitch had been ceased in Finland in 1960s, bitumen was used in road paving instead of coal tar pitch in most countries in Europe [29]. But workers may be exposed to coal tar pitch in repaving old road in recent days.

Aluminum is the third most abundant element in the earth, and is usually present in the form of aluminum silicate in nature. Since Hall and Héroult developed an electrolysis process for aluminum production (Hall-Héroult process) in 1886, almost all aluminum has been produced in this process. In this process, Søderberg anodes are formed from a paste of petroleum coke and coal tar pitch [8]. China is the largest producer of aluminum, followed by Russia, India, United Arab Emirates, and Unites States [30].

Other sources of occupational exposure to PAHs are graphite electrode manufacture [31], diesel engine exhaust [32], manufacture or use of paint containing coal tar [33], and others.

#### Environmental exposure

The air concentration of PAHs varies from 5 ng/m^3^ to 200,000 ng/m^3^ [34, 35]. PAHs in the environment are lower than the workplace, but may spread more widely and cause serious problems in public health. According to the Agency for Toxic Substances and Disease Registry (ATSDR), atmospheric PAHs concentrations in rural and urban areas were 0.02–1.2 ng/m^3^ and 0.15–19.3 ng/m^3^, respectively [12].

Smoking is an important source of PAHs in the environment. An individual who smoke a cigarette will inhale 20–40 ng of benzo(a)pyrene [36]. The sidestream that naturally bloom in burning cigarettes contains a greater amount of PAHs than mainstream [37].

Seventy percent of the non-occupational exposure to PAHs in non-smokers occurs in food [38]. The concentration of PAHs in food varies greatly. The use of charcoal or other kinds of fire to burn meat or barbecues produces significant amount of PAHs [39]. Many foods, such as tea, roasted peanuts, coffee, refined vegetable oil, cereal, and spinach, contain PAHs.

### Standards for PAHs exposure

There is no standard for occupational exposure to specific type of PAHs. Occupational Safety and Health Administration (OSHA) regulated the standard for occupational exposure to coal tar pitch volatiles (CTPVs) and coke oven emissions (COEs) in the Air Contaminants Standard. Coal tar pitch volatiles contains various type of PAHs such as benz(a)anthracene, benzo(b)fluoranthene, chrysene, anthracene, benzo(a)pyrene, phenanthrene, acridine, pyrene, and others. The OSHA permissible exposure limit (PEL) for occupational exposure to CTPVs is 0.2 mg/m^3^ 8 h time weighted average (TWA) [40]. The recommended exposure limit (REL) of National Institute for Occupational Safety and Health (NIOSH) for occupational exposure to CTPVs is 0.1 mg/m^3^ TWA [41], and the threshold limit value (TLV) of ACGIH for occupational exposure to CTPVs is 0.2 mg/m^3^ TWA [42]. The OSHA PEL for occupational exposure to COEs is 0.15 mg/m^3^ [43].

The Korean Ministry of Employment and Labor stipulates the standards for occupational exposure to naphthalene, and there are no standards for other type of PAHs such as benzo(a)pyrene. The standard for occupational exposure to naphthalene is regulated as 10 ppm (50 mg/m^3^) TWA and 15 pm (75 mg/m^3^) short-term exposure limit (STEL). In addition, the standard for occupational exposure to CTPVs is regulated as 0.2 mg/m^3^ TWA in coke making or handling operations, which are high risk for exposure to PAHs (Table 1).

**Table 1:** Standards for occupational exposure to PAHs

	PAHs	Exposure limit	
OSHA	CTPVs	0.2 mg/m^3^	PEL TWA
	COEs	0.15 mg/m^3^	PEL TWA
NIOSH	CTPVs	0.2 mg/m^3^	REL TWA
ACGIH	CTPVs	0.2 mg/m^3^	TLV TWA
MOEL (Korea)	CTPVs	0.2 mg/m^3^	TWA
	Naphthalene	50 mg/m^3^	TWA
		75 mg/m^3^	STEL

*OSHA* Occupational Safety and Health Administration; *NIOSH* National Institute for Occupational Safety and Health; *ACGIH* American Conference of Governmental Industrial Hygienist; *MOEL* Ministry of Employment and Labor; *CTPVs* Coal tar pitch volatiles; *COEs* Coke oven emissions; *PEL* Permissible exposure limits; *TWA* 8 h time-weighted average; TLV Threshold limit value; *STEL* Short-term exposure limits

### Carcinogenicity of PAHs

Specific form or mixture of PAHs can increase the incidence of cancer, and carcinogenic potency of PAHs vary according to the form of PAHs or dose of them [8]. In addition, there are few jobs in which only one form of PAHs is exposed, and most jobs produce various forms of PAHs. Several occupations that can be exposed to PAHs have been classified as carcinogens by IARC [8]. Coal gasification, coke production, chimney sweep, paving and roofing with coal tar pitch, and aluminum production are carcinogenic to humans (group 1), which can cause lung cancer. Coal tar distillation, chimney sweep, and paving and roofing with coal tar pitch are carcinogenic to humans (group 1), which can cause skin cancer except melanoma. Aluminum production is carcinogenic to humans (group 1), which can cause bladder cancer. Paving and roofing with coal tar pitch is also probably carcinogenic to humans (group 2A), which can cause bladder cancer. Carbon electrode manufacture is probably carcinogenic to humans (group 2A), which can cause lung cancer, and creosote is probably carcinogenic to humans (group 2A), which can cause lung and skin cancer except melanoma.

National Toxicology Program (NTP) classified coal tars and coal tar pitches, and COEs as known to human carcinogens (group K) [44]. US Environmental Protection Agency (EPA) classified COEs as human carcinogen (group A) [45]. Coal tar is produced in coal tar distillation process, so coal tars and coal tar pitches classified as group K in NTP is the same as coal tar distillation and paving and roofing with coal tar pitch, classified as group 1 or 2A in IARC. COEs is produced in coke production process, so COEs is the same as coke production, classified as group 1 in IARC (Table 2).

**Table 2:** Carcinogenicity of PAHs

	Classification	Target organ
IARC		
Coal gasification	Group 1	Lung
Coke production	Group 1	Lung
Coal tar distillation	Group 1	Skin (except melanoma)
Chimney sweep	Group 1	Lung, skin (except melanoma)
Paving and roofing with coal tar pitch	Group 1	Lung, skin (except melanoma)
	Group 2A	Bladder
Aluminum production	Group 1	Lung, bladder
Creosote	Group 2A	Lung, skin (except melanoma)
Carbon electrode manufacture	Group 2A	Bladder
NTP		
Coal tars and coal tar pitches	Group K	
Coke oven emissions	Group K	
EPA		
Coal tar pitch volatiles	Group A	

*IARC* International Agency for Research on Cancer, *NTP* National Toxicology Program, *EPA* Environmental Protection Agency, *Group 1* Carcinogenic to humans, *Group 2A* Probably carcinogenic to humans, *Group K* Known to human carcinogens, *Group A* human carcinogen

### Occupational cancer cases in Korea

There have been 29 cases which were evaluated for occupational cancers related PAHs in 2000 to 2014. Among them, 3 cases were recognized as occupational cancers related with PAHs. The first case was a lung cancer in 49 years old man, who was shipbuilding and repair worker. He performed the painting inside the ship for 25 years from 1987, and he used paints containing coal tar until April 2004. The second case was a lung cancer in 59 years old man, who was a heat-treatment worker at an automobile plant. He performed heat-treatment in forging plant for 40 years from 1979, and was exposed to PAHs from incomplete combustion of Bunker C oil and diesel. The third case is a lung cancer in 51-year-old man, who had been involved in the carburizing process from 1988. Carburizing is a heat treatment process in which iron or steel absorbs carbon while the metal is heated in the presence of a carbon-bearing material. He was exposed to PAHs in carburizing process for 23 years.

### Evaluation for work-relatedness and standards for recognition

PAHs are various aromatic hydrocarbons, and there are no standards for occupational exposure to each form of PAHs. In addition, jobs related with PAHs produce various forms of PAHs. So most researches have focused on processes that can be exposed to PAHs, and IARC classified several occupations which can be exposed to PAHs as carcinogens. For these reasons, the evaluation for work-relatedness and standards for recognition of occupational cancers should also focus on occupations that can be exposed to PAHs.

Cancers related with PAHs are lung, bladder, and skin cancers except melanoma. Patients with lung, bladder, or skin cancer except melanoma should have been suspected as occupational cancers associated with PAHs, if they have had the following occupational histories: coal gasification, coke production, coal tar distillation, chimney sweep, paving and roofing with coal tar pitch, aluminum production, creosote, and carbon electrode manufacture. Considering that the latent period of solid cancers is about 10–15 years, if a patient has performed the above occupations for 10–15 years, he (or she) may be diagnosed with occupational cancer.

In Korea, standards for recognition of occupational cancers related with PAHs are following: lung cancers related with more than 10 years exposure to coal tar pitch, lung and skin cancers related with soot exposure, and skin cancers related with more than 10 years exposure to coal tar. These standards should be interpreted considering the occupations that can be exposed to PAHs. “Exposure to coal tar pitch” means occupations containing coal gasification, coke production, job using coal tar pitch, and aluminum production. “Exposure to coal tar” means occupations containing coal tar distillation and job using coal tar pitch. “Soot exposure” occurs during chimney cleaning, but this job does not exist in Korea at present. Soot-like health hazards may be produced in cleaning for fireplace, stove, or boiler, and burning charcoal, so these jobs may be applied in accordance with the soot exposure.

## Conclusions

In Korea, standards for recognition of occupational cancers related with PAHs are lung or skin cancers related with exposure to coal tar pitch, soot, or coal tar. When applying these standards, occupations that can be exposed to PAHs should be focused on. In addition, latent period for solid cancer should be considered. In addition to these occupations, diesel engine combustion and firefighters can be exposed to PAHs. Cancers in these occupations are not covered by the above standards. Cancers in workers engaged with diesel engine combustion or firefighters may be associated with PAHs, so they should be evaluated individually.
